# Utilizing first void urine for high-risk HPV testing for cervical cancer screening in HIV-positive women in Katete, Zambia

**DOI:** 10.1186/s12905-023-02212-7

**Published:** 2023-02-11

**Authors:** Marian Kaoma, Oladapo Olayemi, Mwila Hilton Mwaba, Kapembwa Sikwewa

**Affiliations:** 1 Department of Obstetrics and Gynaecology, The Pan African University for Life and Earth Sciences Institute (Including Health and Agriculture), University of Ibadan, Ibadan, Nigeria; 2 Department of Obstetrics and Gynaecology, University College Hospital, University of Ibadan, Ibadan, Nigeria; 3 Department of Basic Medical Sciences, Michael Chilufya Sata School of Medicine, The Copperbelt University, Ndola, Zambia; 4 Laboratory Department, St Francis’ Hospital, Katete, Zambia

**Keywords:** First void urine, High-risk, Human papillomavirus, Zambia

## Abstract

**Background:**

The World Health Organization targets to screen 70% of women worldwide twice for cervical cancer by the year 2030, first by age of 35, and again by the age of 45. However, with the current low screening coverage in many developing countries, this may not be achieved because the invasive sampling method is unacceptable to some. In Zambia, for instance, despite the availability of free cervical cancer screening through the establishment of the Cervical Cancer Prevention Programme, some women are still reluctant to go for screening. First void urine sampling is non-invasive and thus has the potential to increase screening coverage. We aimed to determine the performance of first void urine for high-risk human papillomavirus DNA detection, the prevalence of high-risk HPV, and the acceptability of first void urine sampling.

**Materials and method:**

A comparative cross-sectional study was conducted among 100 HIV- infected women at St Francis’ Hospital in Zambia, attending the routine HIV/AIDS services and cervical cancer screening. 17 mL of first void urine sample collected by each participant was immediately mixed with 3 mL of 0.5 M EDTA preservative solution before cervical sample collection by the clinician. For testing, 2 mL of first void urine and 1 mL of the cervical sample were tested using the GeneXpert platform. An interview-based questionnaire was used to gather data on the acceptability of first void urine sampling. Data was analyzed using Stata version 17.

**Results:**

The mean age of the participants was 42.58 years (95% CI 40.98–44.19; SD 8.01). High-risk HPV prevalence was 34% (95% CI 24%-43.9%) in both cervical and first void urine samples. Sensitivity and specificity were 84.8% (95% CI 68.1%–94.9%) and 92.3% (83%–97.5%), respectively. There was 89.80% agreement between the samples (κ = 0.77; 95% CI 0.64–0.91). First void urine sampling was highly accepted.

**Conclusion:**

High-risk HPV DNA can be detected in first void urine samples using the GeneXpert, with a substantial agreement with cervical samples. An affordable preservative such as Ethylenediamine tetraacetic acid can prevent DNA degradation. With optimization, first void urine sampling has the potential to increase screening coverage.

**Supplementary Information:**

The online version contains supplementary material available at 10.1186/s12905-023-02212-7.

## Background

Cervical cancer is reported to be the fourth most common cancer affecting women worldwide and Africa’s second most common cancer [1, 2]. Developing countries account for about 70% of the global burden of this disease. Approximately 85% of cervical cancer deaths occur in developing countries, with the highest regional occurrence in Sub-Saharan Africa, where about 59% of women and girls are living with HIV [3, 4]. In 2020, there were about 604,127 new cases and 341,831 deaths globally, with about 76, 745 of these deaths occurring in Africa alone [2]. These deaths are unnecessary because cervical cancer can be prevented [2, 5]. By 2040, the burden of cervical cancer is expected to double what was reported in 2020 [6]. Therefore, if cervical cancer screening is to gain success in many parts of the world, target populations must effectively participate in regular screening [7]. With more than 95% of cervical cancer cases occurring due to persistent infection with high-risk human papillomaviruses, the World Health Organization (WHO) recommends human papillomavirus DNA testing for screening [8]. Screening by HPV detection provides about 60–70% protection against invasive cervical cancer, and also allows for self-sampling [9]. HIV-infected women are about six times more like to develop cervical cancer compared to their HIV-negative counterparts [10]. With the efforts made to expand HIV treatment to the people that need it, there is a need to make certain that these women’s lives are not cut short by cervical cancer when it can be prevented [10]. The integration of cervical cancer screening into HIV/AIDS services is a good opportunity to increase screening coverage in this population. However, if this service is not well-utilized, cervical cancer cases will continue to increase.

According to the 90–70–90 global strategy, the World Health Organization targets to screen 70% of women worldwide for cervical cancer twice by the year 2030, first by the age of 35 and again by the age of 45 [10]. However, cervical and vaginal sampling are invasive and therefore some women are not encouraged to go for screening. Factors contributing to the poor acceptability of cervical and vaginal sampling by some women include fear of pain, embarrassment, and religious and cultural beliefs. Therefore, we need to explore alternative methods of sampling in order to improve screening coverage [11]. Urine sampling provides an opportunity to expand cervical cancer screening coverage by reaching populations reluctant to undergo cervical or vaginal sampling [12]. The first 20 mL of urine flush (first void) is contaminated with exfoliated cells from the cervix and vagina, and thus provides an acceptable sample for HPV DNA testing [4, 12, 13].

Cervical cancer is the most prevalent cancer affecting females in Zambia and the leading cause of death in this population. Despite the introduction of free screening through the establishment of the Cervical Cancer Prevention Programme in Zambia (CCPPZ) in 2006, there are still women reluctant to go for screening. Zambia recorded the third-highest incident rate of cervical cancer in the year 2018, with about 66.4 new cases per 100, 000 women. Later in 2020, the prevalence of cervical cancer in Zambia was 40.2% [14]. This represented one of the world’s highest disease burden, suggesting an urgent need for more effective preventative measures in the country [14]. Only a few studies have assessed the performance of first void urine sampling for HPV testing using the GeneXpert platform [11, 15, 16]. To date, there has been no study done in Zambia to assess the performance of first void urine for HPV DNA testing. Human papillomavirus detection in first void urine can help increase screening coverage by reaching women reluctant to undergo vaginal self-sampling or clinician-based cervical sampling [17]. Therefore, we aimed to determine the performance of first void urine sampling for high-risk HPV DNA testing as an alternative sample for cervical cancer screening using the clinician-collected cervical samples as reference using the GeneXpert platform. We also assessed the acceptability of first void urine sampling for cervical cancer screening.

## Methods

### Study design and setting

A comparative cross-sectional study was conducted at St Francis’ Hospital Cervical cancer screening Clinic and Laboratory department in Katete District, Zambia between April and May 2022. Katete is a rural district located along Great East road, approximately 490 km from Lusaka, the capital city of Zambia. St Francis’ Hospital is a second-level mission hospital, founded in 1948 by the Anglican Church and is currently jointly managed by the Anglican and Catholic Churches. It is the largest health facility in Katete district, serving as a referral facility for patients in the Eastern province of Zambia, and also provides services to patients coming from the neighbouring Mozambique and Malawi [18].

### Study population

The study population comprised of HIV-infected women aged 25–59 years, non-pregnant, non-menstruating, unvaccinated for HPV, sexually active, had no sexual intercourse within 48 h, and had not urinated at least 1 h before arriving for screening. Women excluded were those outside the age group (25–59 years), menstruating, pregnant, with no history of sexual intercourse, vaccinated for HPV, had cervical cancer, were not sufficiently healthy to participate, and those unwilling to participate. The participants were recruited consecutively as they came for antiretroviral therapy services and cervical cancer screening.

### Sample collection and storage

**Fig. 1:**
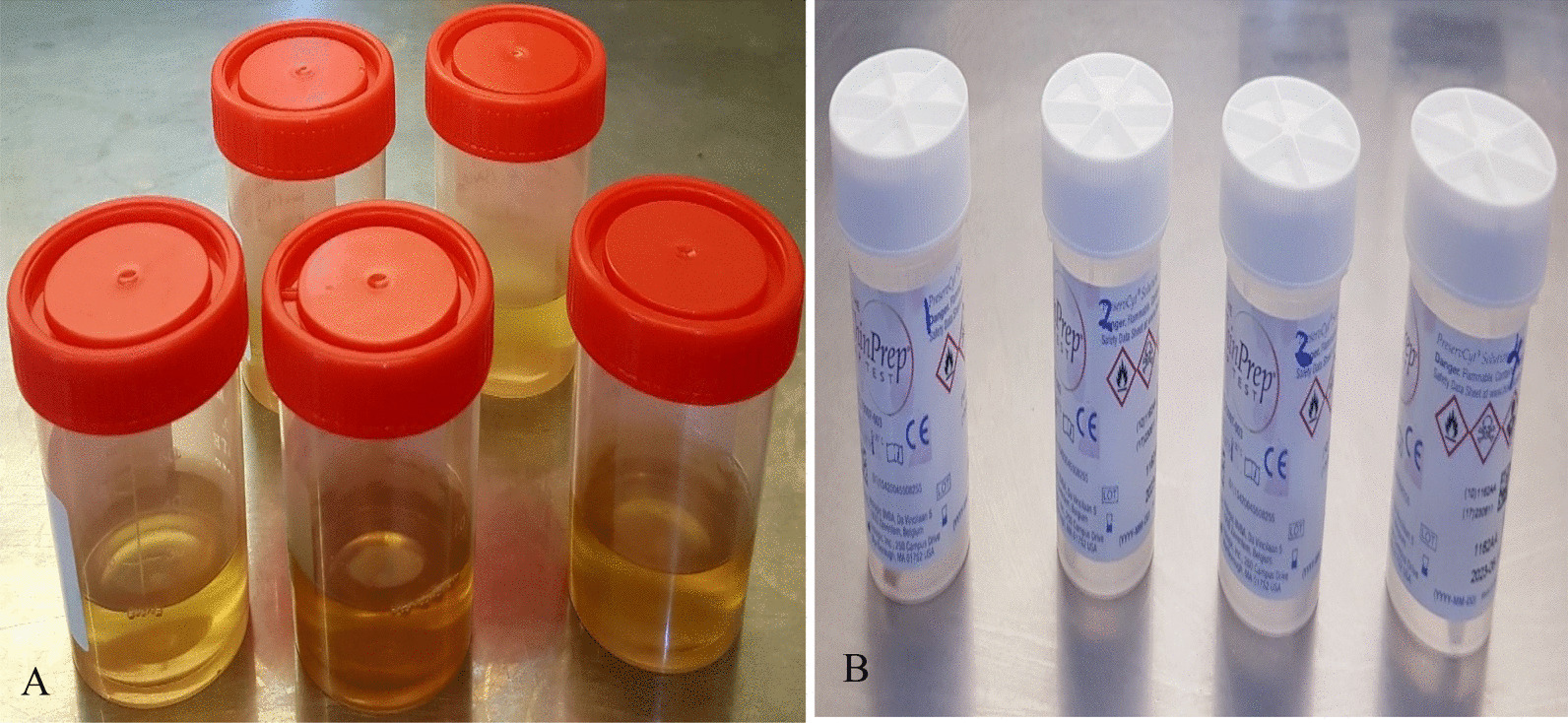
**a**, **b** Selected examples of EDTA preserved first void urine samples and cervical samples in preservCyt solution

Paired first void urine and cervical samples were collected consecutively from 100 HIV- infected women. The women were verbally instructed to collect 17 mL of the first flush of urine (marked on the container) while squatting into a sterile 50 mL sterile container without cleaning their genital area prior to urination. The urine samples were immediately mixed with 3 mL of 0.5 M EDTA preservative solution (pH 8) by gently shaking four times, and then the samples were refrigerated at 4 °C before processing (Fig. 1a). As per routine, cervical samples were collected by a registered nurse using a Copan swab (FLOQSwab™) by rotating it four times at the cervix for 20 s while the women lay in a lithotomy position. The cervical swab was then rinsed in 20 mL of ThinPrep preservCyt solution (Hologic, Marlborough) and sent to the laboratory for processing (Fig. 1b). Following sample collection, an interview-based questionnaire on the acceptability of sample collection was administered.

### HPV DNA testing

The cervical sample in ThinPrep preservCyt solution was mixed, and 1 mL was pipetted into the  HPV cartridge. The cartridge was then slotted into the Xpert system for sample processing. With blinding results from the cervical sample, the first void urine sample was mixed by gently inverting the container 8–10 times. Then 2 mL was pipetted into the cartridge and loaded into the Xpert system for processing. Urine samples not tested on the same day of collection were stored in the refrigerator at 4 °C, and tested the following day. All women with positive results from cervical samples underwent visual assessment for treatment (VAT). Those that needed treatment with conditions that could be treated at St Francis’ Hospital were treated there.

### Acceptability questionnaire

An interview-based questionnaire with closed-ended questions was administered in English or Chichewa, the local language of Katete district. The privacy of the participants was well-respected. Questionnaire responses were grouped as “Yes”, “No”, and “Not sure”. The questionnaire addressed whether the women encountered any difficulty in collecting first void urine, if they found first void urine sampling more comfortable than cervical sampling, and which method they would prefer in future alone for sample collection (see Additional file 1).

### Statistical analysis

Only women with valid results for both first void urine and cervical samples were included in the statistical analysis. Data was analyzed using Stata version 17, and additional graphs were generated using Microsoft Excel 2016. Sensitivity, specificity, predictive values, and likelihood ratios (LR + and LR −) were calculated at 95% confidence interval. Concordance between first void urine and cervical samples was interpreted as slight (κ < 0.20), weak (κ = 0.21–0.40), moderate (κ = 0.41–0.60), substantial (κ = 0.61–0.80), near perfect (κ = 0.81–0.99) or perfect (κ = 1.00). We used McNemar’s test to assess for differences between the paired proportions. Results were statistically significant with a *p* value < 0.05. The overall performance of the test was determined by constructing a receiver operating characteristic (ROC) curve. The area under the curve was interpreted as excellent (0.9–1.0), very good (0.8–0.9), good (0.7–0.8), sufficient (0.6–0.7), bad (0.5–0.6), and not useful (< 0.5) [19]. Acceptability and preference of first void urine versus cervical sampling were evaluated as proportions at 95% confidence intervals. Chi-square goodness of fit was used to assess whether there was a significant difference in the proportions of responses, with *p* < 0.05 indicating a significant difference. We also tested whether acceptability and preference differed significantly between different age groups using the chi-square test, where a *p* value < 0.05 indicated there was a significant difference.

## Results

### Demographic characteristics of the participants

Between April and May, 2022, paired first void urine and cervical samples were collected from 100 HIV-infected women. Ninety-eight women having valid results for both first void urine and cervical samples were considered for analysis. Two women (2%) having invalid first void urine results were excluded from the analysis. The mean age of the participants was 42.58 years (95% CI 40.98–44.19; SD: 8.01). The participants’ demographic characteristics are presented in Additional file 2. Almost half of the women were married (48.98%), and more than half (65.31%) stopped their education in primary level. The majority of the women were either housewives or dependents with no source of income (34.69%).

### Prevalence of high-risk Human papillomavirus

The prevalence of high-risk HPV was 34% (95% CI 24%—43.9%) for both cervical and first void urine samples. However, we found 10 discordant results, 5 first void urine samples were negative for high-risk HPV, but cervical samples were positive, and 5 were positive for high-risk HPV DNA but negative for cervical samples (Table 1).

**Table 1:** 2 × 2 table for first void urine versus cervical samples for high-risk HPV DNA test

		Cervical samples		
		Positive	Negative	Total
**First void urine samples**	Positive	28	5	33
	Negative	5	60	65
	Total	33	65	98

The high-risk HPV prevalence was highest in the age group 32–45 years (9.18%). The age group 25–31 years had the lowest prevalence of 3.06% in cervical samples and 4.08% in urine samples. We found that the age group of the participants was not significantly associated with hrHPV infection for both cervical (χ2 = 1.92; *P* = 0.588) and first void urine (χ2 = 2.52; *P* = 0.472).

**Fig. 2:**
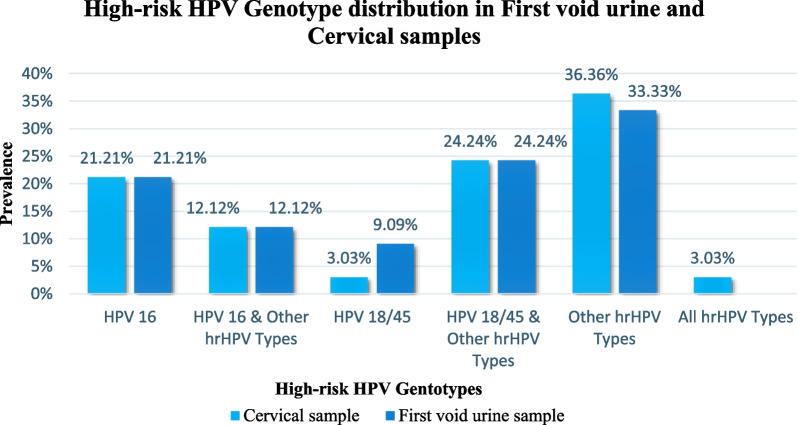
High-risk HPV genotype distribution in HIV-positive women in Katete, Zambia

The most prevalent high-risk HPV genotypes were those classified as ‘Other high-risk HPV types’ by the GeneXpert platform (HPV 33, 31, 35, 39, 51, 52, 56, 59, 66, and 68), with a prevalence of 36.36% in cervical samples and 33.33% in first void urine samples. Co-infection with a combination of hrHPV 18/45 & Other hrHPV genotypes (except HPV 16, 18 & 45) had the second highest prevalence of 24.24% in both samples. HPV 16 was the third most prevalent genotype detected, with a prevalence of 21.21% in both samples. All groups of  hrHPV were represented in one woman’s sample (Fig. 2). There was no statistically significant difference in high-risk HPV genotype distribution in first void urine and cervical samples (McNemar’s test: *P* –value = 0.706).

### Assessment of clinical performance

The sensitivity and specificity of hrHPV DNA detection in first void urine were 84.8% (95% CI 68.1%–94.9%) and 92.3% (95% CI 83%–97.5%), respectively. The positive predictive value (PPV) was 84.8% (95% CI 68.1%–94.9%) and the negative predictive value (NPV) was 92.3% (95% CI 83%–97.5%). We also found positive and negative likelihood ratios of 11 (95% CI 4.69–25.9) and 0.16 (95% CI 0.07–0.37), respectively. The odds ratio was 67.2 (95% CI 18.4–245) (Table 2). There was no significant difference between the first void urine and cervical sample results (McNemar’s test: *P-*value = 1.000).

**Table 2:** Calculated values of sensitivity, specificity, ROC area, predictive values, likelihood ratios and odds ratio

		95% confidence interval		
Sensitivity	Pr(+/A)	84.8%	68.1%	94.9%
Specificity	Pr(−/N)	92.3%	83%	97.5%
ROC area	(Sens. + Spec.)/2	.886	.816	.956
Positive predictive value	Pr(A/+)	84.8%	68.1%	94.9%
Negative predictive value	Pr(N/−)	92.3%	83%	97.5%
Likelihood ratio (+)	Pr(+/A)/Pr(+/N)	11	4.69	25.9
Likelihood ratio (−)	Pr(−/A)/Pr(−/N)	.164	.073	.369
Odds ratio	LR(+)/LR(−)	67.2	18.4	245

The overall performance of first void urine for high-risk HPV DNA detection was visualized using a receiver operating curve by determining the area under it. The area under the curve was found to be 0.89 (95% CI 0.82–0.96) with a standard error of 0.04.

### Concordance between samples

There was substantial agreement between the first void urine and cervical samples for hrHPV DNA detection. The overall agreement was 89.80% (95% CI 82.2%-94.4%) and the kappa statistic was 0.77 (95% CI 0.64- 0.91), with a *P*-value < 0.001. Positive agreement was 84.8% (95% CI 69.1%–93.4%) and negative agreement was 92.3% (95% CI 83.2%–96.7%). However, there was 10.2% (10/98) discordance between the two samples. We also determined the agreement of genotypes in urine and cercical samples. Overall, there was good agreement between genotypes in first void urine and cervical samples of 89.29% (κ = 0.86; 95% CI 0.82—0.95), except for hrHPV 18/45, with a percentage agreement of only 33.33% (κ = 0.00), and in one sample in which all hrHPV genotypes were detected in the cervical sample but not in the urine sample (Table 3).

**Table 3:** High-risk HPV genotypes detected in cervical and first void urine samples

High-risk HPV Genotype	Cervical sample n (%)	First void urine n (%)	Agreement (%)	Kappa (κ)
16	7 (21.21)	7 (21.21)	100.00	1.00 (1.00–1.00)
18/45	1 (3.030)	3 (9.09)	33.33	0.00
Other hrHPV	12 (36.36)	11 (33.33)	91.67	0.833 (0.60–1.00)
HPV 16 & Other hrHPV	4 (12.12)	4 (12.12)	100.00	1.00 (1.00–1.00)
HPV 18/45 & Other hrHPV	8 (24.24)	8 (24.24)	100.00	1.00 (1.00–1.00)
All hrHPV types	1 (3.03)	0 (0.00)	–	–

The kappa value was interpreted as follows: no agreement (κ ≤ 0), none to slight agreement (κ = 0.01–0.20), fair agreement (κ = 0.21–0.40), moderate agreement (κ = 0.41–0.60), substantial agreement (κ = 0.61–0.80), near perfect agreement (κ = 0.81–0.99) and perfect agreement (κ = 1.00)

### Acceptability of urine sampling

Majority of the women (87.78%; 95% CI 80.74%–93.72%) had been screened before, only 11.22% (95% CI 5.73%–19.20%) were new clients. More than half (55.10%; 95% CI 45.06%–64.75%) of them said they did not feel embarrassed showing their genitalia to the clinician for cervical sample collection, while 39 (39.80%) found it to be embarrassing. First void urine sampling was more comfortable than cervical sampling for 90.82% (95% CI 83.16%–95.19%) of the women, while only 4.08% (95% CI 1.52%–10.49%) said cervical sampling was more comfortable for them. Only 9.18% (95% CI 4.81%–16.84%) stated they found first void urine collection difficult. About 87.76% (95% CI 79.54%–92.96%) of the women said they would prefer urine sampling alone in future for cervical cancer screening. Preference for first void urine sampling did not differ significantly among different age groups, with *P-*value of 0.68 (see Additional file 3).

## Discussion

HIV-infected women are among the underscreened populations despite having a higher risk of developing cervical cancer than their HIV-negative counterparts [20, 21]. In Zambia, efforts have been made to increase screening coverage by integrating cervical cancer screening in HIV/AIDS services and encouraging vaginal self-sampling. However, these services still remain underutilized because the invasive sampling method for HPV testing  is not acceptable to all. Urine as a liquid biopsy has the potential to remove barriers to screening due to its non-invasiveness and ease of collection [22]. In this study, we aimed to assess the performance of first void urine for the detection of high-risk HPV DNA using the clinician collected cervical samples as reference using the GeneXpert platform.

Our study confirms that HPV DNA can be detected in first void urine as reported by several studies. However, different results have been reported [12, 13, 16, 17, 23, 24]. In this study, we found substantial agreement between first void urine and clinician-collected cervical samples with prevalence of 34% (95% CI 24%–43.9%) for both samples, and infections with hrHPV types classified as ‘Other hrHPV types’ by the GeneXpert platform being more common. Sensitivity and specificity were 84.8% (95% CI 68.1%–94.9%) and 92.3% (95% CI 83%–97.5%), respectively. The differences in the results of this study with has been reported elsewhere could be due to variations in the study settings, population, sampling procedures, conditions of storage, urine volume collected/amount used for testing, and assays used [13, 25]. Most studies were conducted in a colposcopy clinic among women with cervical abnormalities and not in a screening population.  Therefore, this might explain the  lower specificities but higher sensitivities reported in some studies considering specificity decreases with an increase in disease prevalence, while sensitivity increases. In addition, sensitivity increases when using a highly sensitive assay [12, 26]. However, despite our sensitivity being higher than what has been  reported by some other authors [16, 17, 24], there is still a need for optimization in order to reduce false negatives considering the rationale for the detection of HPV DNA in urine [17]. Before urine sampling can be used as an alternative sample for cervical cancer screening, there is a need for standardization of methods, and CIN prediction also needs to be assessed [12]. The negative hrHPV results for the first void urine when cervical samples were positive might have been due to low numbers of HPV- infected cells exfoliated from the cervix. This low cellular yield of DNA in urine might have also led to getting 2 invalid first void urine test results. The possible explanation for detecting hrHPV DNA in urine samples when cervical samples were negative is that the hrHPV detected in the urine samples might have been from the vaginal or vulva epithelial tissues, as they are also susceptible to HPV infection [27, 28].

First void urine sampling was found to be highly acceptable by the women, even with just the use of a simple urine cup. Ninety-one percent of the women found it to be more comfortable than cervical sampling (95% CI 83.16%–95.19%), with almost 90% (87.76%; 95% CI 79.54%–92.96%) of them stating they would prefer urine sampling in future for HPV testing for cervical cancer screening. This was in line with previous studies in which urine sampling was preferred over cervical or vaginal sampling for either collection at home or at the health facility [11, 15, 17, 29, 30]. With all the women in our study stating they had at least heard something about cervical cancer, it was evident that cervical cancer outreach programmes by the Zambian Ministry of Health are effective in raising awareness. Therefore, the lack of participation by some women may not be due to a lack of knowledge, but fear, embarrassment, and lack of acceptability of the conventional sampling method. With the majority of the women in this study (55.10%) stating they did not feel embarrassed during the clinician-based cervical sampling, this may not be the case among the non-attendees [31, 32].

### Strengths and limitations

Our study had several strengths. We conducted the study in a primary cervical screening population where urine sampling would most likely be useful. A clinically validated and highly sensitive HPV assay (GeneXpert) was used, and urine samples were tested with blinding of cervical results. We used an affordable urine collection container and preservative that is more practical in low-resource settings as reported by Hernandez-Lopez et al. [23]. Most of the previous studies have used specially designed urine collection containers and preservatives that are costly for developing countries [32]. However, EDTA solution is inexpensive and has been reported to prevent HPV DNA degradation in urine [23]. As suggested by Marcus et al. [16], we used 2 mL of first void urine for testing to help improve HPV DNA detection. To our best knowledge, this is the first study to assess the performance of first void urine preserved with EDTA solution using the GeneXpert platform in a screening population. The GeneXpert has a rapid turnaround time of 1 h and is suitable for both low-and high-resource settings [16].

However, our study also had some limitations that need to be acknowledged. Firstly, we had a small sample size. Secondly, the GeneXpert platform only carries out partial genotyping and therefore, we could not determine which specific type of hrHPV  was most prevalent. Lastly, our study only showed the performance of first void urine in comparison to cervical samples for HPV DNA detection using the GeneXpert platform. There is need for future studies to determine the performance of first void urine for HPV DNA detection using the GeneXpert platform with the assessment of CIN2 + detection.

### Recommendations

To improve hrHPV DNA detection in first void urine, there is a need for an increased interval between previous urination and urine sampling as this would result in an increased accumulation of exfoliated debris from the cervix and thus an increase in HPV DNA in urine [3]. In this study, first void urine was collected at least 1 h after the previous urination as reported by other authors [3, 15]. However, increasing the interval between two urinations to at least 2 h could result in improved HPV DNA detection due to increased HPV DNA concentration as a result of an increase in the accumulation of mucus and secretions from the cervix and vagina that are captured in first void urine [12, 15]. Optimal conditions for longer storage of EDTA preserved first void urine should be explored to allow for home collection of samples [23]. Collection containers designed to capture a standardized volume of first void urine, prefilled with a preservative solution can be used to reduce on sampling errors [3]. In addition, future studies should carry out hrHPV genotyping to determine the actual prevalence of different hrHPV types and also investigate the performance of first void urine with the assessment of CIN2 + detection.

## Conclusions

This study showed that high-risk HPV DNA can be detected in first void urine using the GeneXpert platform, with substantial agreement with cervical samples. There was high acceptability of first void urine sampling for cervical cancer screening in HIV-positive women. Therefore, with optimization, first void urine sampling has the potential to increase participation in cervical cancer screening programmes due to its non-invasiveness, especially in developing countries where there is a high burden of cervical cancer. However, if urine sampling is to be used as an alternative sample for hrHPV DNA testing, there is still a need for further optimization and standardization to make the results more comparable to those obtained from cervical samples.

## Supplementary Information

**Additional file 1.** Questionnaire on the acceptability of first void urine sampling by the participating women

**Additional file 2.** Table of Demographic characteristics of the study participants attending HIV/AIDS services and cervical cancer clinic at St Francis’ Hospital, Zambia (n = 98)

**Additional file 3.** Acceptability responses on first void urine sampling for cervical cancer screening

## Data Availability

The dataset used in the  study is not publicly available but might be made available upon reasonable request from the corresponding author.

## Abbreviations

CCPPZ: Cervical cancer prevention programme in Zambia
CI: Confidence interval
DNA: Deoxyribonucleic acid
EDTA: Ethylenediamine tetraacetic acid
HIV: Human immunodeficiency virus
HPV: Human papillomavirus
HSIL+: High-grade squamous intraepithelial lesion
hrHPV: High-risk human papillomavirus
κ: Kappa value
SD: Standard deviation
VAT: Visual assessment for treatment
WHO: World Health Organization
