# Supplementary material for: Utilizing first void urine for high-risk HPV testing for cervical cancer screening in HIV-positive women in Katete, Zambia
Source: BMC Womens Health. 2023 Feb 11;23:62. doi: 10.1186/s12905-023-02212-7 (PMC9922459; doi:10.1186/s12905-023-02212-7)
Supplement: Supplementary file 1 — Additional file 1. Questionnaire on the acceptability of first void urine sampling by the participating women [file 12905_2023_2212_MOESM1_ESM.docx]

**QUESTIONNAIRE**

Please take note that all information given will be kept confidential.

**SECTION A: BIOGRAPHICAL INFORMATION**

1. Age (in years) ………………………………………………………......................
2. Place of residence…………………………………………………………………..
3. Marital status
   1. Single [ ] b. Married [ ] c. Divorced [ ] d. Widowed [ ] e. Separated [ ]
4. Educational level
   1. Primary [ ] b. Secondary [ ] c. Tertiary [ ] d. None [ ]
5. Religion
   1. Christian [ ] b. Muslim [ ] iii. Others [ ] ………………….(specify)
6. Occupation………………………………………..

| **SECTION B: KNOWLEDGE ABOUT CERVICAL CANCER** | | | | |
| --- | --- | --- | --- | --- |
| **No.** | **QUESTIONS** | **RESPONSE** | | |
|  |  | **YES** | **NO** | **NOT SURE** |
| 1. | Have you heard about cervical cancer? |  |  |  |
| 2. | Do you know any risk factor for cervical cancer? |  |  |  |
| 3. | Have you been screened before? |  |  |  |

| **SECTION C: ACCEPTABILITY OF FIRST VOID URINE SAMPLING** | | | | |
| --- | --- | --- | --- | --- |
| **No.** | **QUESTIONS** | **RESPONSE** | | |
|  |  | **YES** | **NO** | **NOT SURE** |
| 1. | Did you encounter any difficulty in collecting the first void urine? |  |  |  |
| 2. | Did you feel embarrassed showing your genitalia to the clinician for cervical sample collection? |  |  |  |
| 3. | Did you find first void urine sample collection more comfortable than cervical sampling? |  |  |  |
| 4. | Overall first void urine sampling is better than cervical sampling. |  |  |  |
| 5. | If I could choose, I would prefer urine sampling alone in the future for cervical cancer screening rather than cervical sampling. |  |  |  |
