# Supplementary material for: Utilizing first void urine for high-risk HPV testing for cervical cancer screening in HIV-positive women in Katete, Zambia
Source: BMC Womens Health. 2023 Feb 11;23:62. doi: 10.1186/s12905-023-02212-7 (PMC9922459; doi:10.1186/s12905-023-02212-7)
Supplement: Supplementary file 2 — Additional file 2. Table of Demographic characteristics of the study participants attending HIV/AIDS services and cervical cancer clinic at St Francis’ Hospital, Zambia (n = 98) [file 12905_2023_2212_MOESM2_ESM.docx]

**Table S1**: Demographic characteristics of study participants attending HIV/AIDS services and cervical cancer clinic at St Francis’ Hospital, Zambia (n=98)

| Variable Frequency Percentage |
| --- |
| Age Range  25-31 8 8.16  32-38 23 23.47  39-45 36 36.73  46-52 19 19.39  53-59 12 12.24 |
| Marital Status  Single 6 6.12  Married 48 48.98  Separated 7 7.14  Divorced 17 17.35  Widowed 20 20.41 |
| Religion  Christian 94 95.92  Muslim 4 4.08 |
| Level of Education  None 17 17.35  Primary 64 65.31  Secondary 12 12.24  Tertiary 5 5.10 |
| Occupation  Teacher 5 5.10  Receptionist 1 1.02  Business woman 26 26.53  Farmer 32 32.65  None 34 34.69 |
