# Supplementary material for: Utilizing first void urine for high-risk HPV testing for cervical cancer screening in HIV-positive women in Katete, Zambia
Source: BMC Womens Health. 2023 Feb 11;23:62. doi: 10.1186/s12905-023-02212-7 (PMC9922459; doi:10.1186/s12905-023-02212-7)
Supplement: Supplementary file 3 — Additional file 3. Acceptability responses on first void urine sampling for cervical cancer screening [file 12905_2023_2212_MOESM3_ESM.docx]

**Table S2**: Acceptability responses on first void urine sampling for cervical cancer screening

| Response (%; 95% CI)  Yes No Not sure *P*-value |
| --- |
| Heard about cervical cancer 100(96.31-100.00) <0.001  Knows any risk 47.96(37.76-58.29) 23.47(15.50-33.11) 28.57(19.90-38.58) 0.007  factor for cervical cancer  Been screened before using 88.78(80.80-94.26) 11.22(5.74-19.20) <0.001  any method  Encountered difficulty when 9.18(4.29-16.72) 90.82(83.28-95.71) <0.001  collecting first void urine  Clinician cervical sample 39.80(30.04-50.18) 55.10(44.72-65.17) 5.10(1.68-11.51) <0.001  collection is embarrassing  Urine sampling is more 90.82(83.28-95.71) 4.08(1.12-10.12) 5.10(1.68-11.51) <0.001  comfortable than cervical  sampling  Overall urine sampling is 80.61(71.39-87.90) 4.08(1.12-10.12) 15.31(8.83-23.99) <0.001  better than cervical sampling  Preference for urine sampling 87.76(79.59-93.51) 3.06(0.64-8.69) 9.18(4.29-16.72) <0.001  rather than cervical  sampling |

For the chi-square test for goodness of fit, a *P-*value **<0.05** indicates a significant difference in the proportions of responses among the women.
